# The Role of PTEN Loss in Immune Escape, Melanoma Prognosis and Therapy Response

**DOI:** 10.3390/cancers12030742

**Published:** 2020-03-21

**Authors:** Rita Cabrita, Shamik Mitra, Adriana Sanna, Henrik Ekedahl, Kristina Lövgren, Håkan Olsson, Christian Ingvar, Karolin Isaksson, Martin Lauss, Ana Carneiro, Göran Jönsson

**Affiliations:** 1 Division of Oncology, Department of Clinical Sciences, Lund University, 22381 Lund, Sweden; rita.cabrita@med.lu.se (R.C.); shamik.mitra@med.lu.se (S.M.); adriana.sanna@med.lu.se (A.S.); kristina.lovgren@med.lu.se (K.L.); hakan.olsson@med.lu.se (H.O.); martin.lauss@med.lu.se (M.L.); ana.carneiro@med.lu.se (A.C.); 2 Department of Oncology, Skåne University Hospital, 22185 Lund, Sweden; henrik.ekedahl@med.lu.se; 3 Department of Surgery, Skåne University Hospital, 22185 Lund, Sweden; christian.ingvar@med.lu.se; 4 Department of Surgery, Department of Clinical Sciences, Lund University, 22185 Lund, Sweden; karolin.isaksson@med.lu.se; 5 Department of Surgery, Central Hospital Kristanstad, 29133 Kristainstad, Sweden

**Keywords:** melanoma, PTEN, immune evasion

## Abstract

Checkpoint blockade therapies have changed the clinical management of metastatic melanoma patients considerably, showing survival benefits. Despite the clinical success, not all patients respond to treatment or they develop resistance. Although there are several treatment predictive biomarkers, understanding therapy resistance and the mechanisms of tumor immune evasion is crucial to increase the frequency of patients benefiting from treatment. The *PTEN* gene is thought to promote immune evasion and is frequently mutated in cancer and melanoma. Another feature of melanoma tumors that may affect the capacity of escaping T-cell recognition is melanoma cell dedifferentiation characterized by decreased expression of the microphtalmia-associated transcription factor (*MITF*) gene. In this study, we have explored the role of PTEN in prognosis, therapy response, and immune escape in the context of *MITF* expression using immunostaining and genomic data from a large cohort of metastatic melanoma. We confirmed in our cohort that PTEN alterations promote immune evasion highlighted by decreased frequency of T-cell infiltration in such tumors, resulting in a worse patient survival. More importantly, our results suggest that dedifferentiated PTEN negative melanoma tumors have poor patient outcome, no T-cell infiltration, and transcriptional properties rendering them resistant to targeted- and immuno-therapy.

## 1. Introduction

Melanoma is one of the most immunogenic tumor diseases with known cases of spontaneous tumor regression and the frequent presence of tumor infiltrated lymphocytes (TILs). For this reason, melanoma cells provide a suitable model to investigate the molecular crosstalk of cancer cells with cells of the immune system. Advances in this field have allowed the development of efficient therapeutic strategies and a major revolution in the clinical management of metastatic melanoma patients with the introduction of immune checkpoint blockade (ICB) as the primary choice of therapy [1]. The main ICB modalities used in the clinical setting are targeting immune checkpoint molecules, such as CTLA-4 and PD1, in order to prevent the T-cell inhibitory signals mediated by these molecules and thus boost the immune response against the tumor cells. Although clinical benefit is frequent, still, about 60% of patients develop primary resistance, whereas others experience initial clinical response and later on develop secondary resistance [2,3]. Both types of resistance are presumed to be caused by the mechanisms of immune evasion. These mechanisms can be initiated by, among other signaling pathways, MHC molecule downregulation or oncogenic pathways such as the PI3K or the WNT-beta-catenin pathway [4]. On the other hand, *MITF*, a melanoma lineage-specific gene, is also an important player in the immunogenicity of the disease. This gene regulates the expression of pigmentation related molecules, which are recognized by T-cells, thus contributing to the highly immunogenic nature of melanoma [5]. Several studies have shown that the MITF^low^ cells are dedifferentiated, invasive, and apoptosis-resistant [6,7,8], which gives them the ability to survive harsh conditions, such as under targeted and/or immunotherapy agents [5]. Given the fact that melanomas, under harsh conditions, can become enriched in MITF^low^ cells and that these are more capable of escaping T-cell recognition, loss of MITF may be a mechanism for tumor cells to evade the immune system [9]. Moreover, immune evasion mechanisms are activated either by oncogenic gain-of-function, such as CTNNB1 (which encodes for the protein beta-catenin), or loss-of-function of tumor suppressor genes, such as *PTEN* [10]. Several studies showed that beta-catenin-positive tumors had minimal T-cell infiltration and were resistant to ICB [11]. Although it remains to be shown whether the lack of beta-catenin pathway activation contributes to the clinical benefit of anti-PD1 therapy, targeting this pathway might be a potential strategy to improve ICB response [10]. *PTEN* belonging to the PI3K signaling pathway is frequently mutated and associated with a lack of T-cell infiltration in melanoma [4,12]. Although the mechanisms by which *PTEN* deletion might promote immune evasion are incompletely understood, recent studies in melanomas with PTEN loss have motivated clinical trials of specific PI3K inhibitors in combination with ICB [10,13]. In this study, we have explored the role of PTEN in prognosis, therapy response, and immune escape in the context of MITF expression in melanoma. Our results suggest that, in particular, MITF- and PTEN-negative melanoma tumors have molecular properties rendering them resistant to targeted- and immuno-therapy.

## 2. Results

### 2.1. PTEN Protein Expression in Metastatic Melanoma

**Figure 1:**
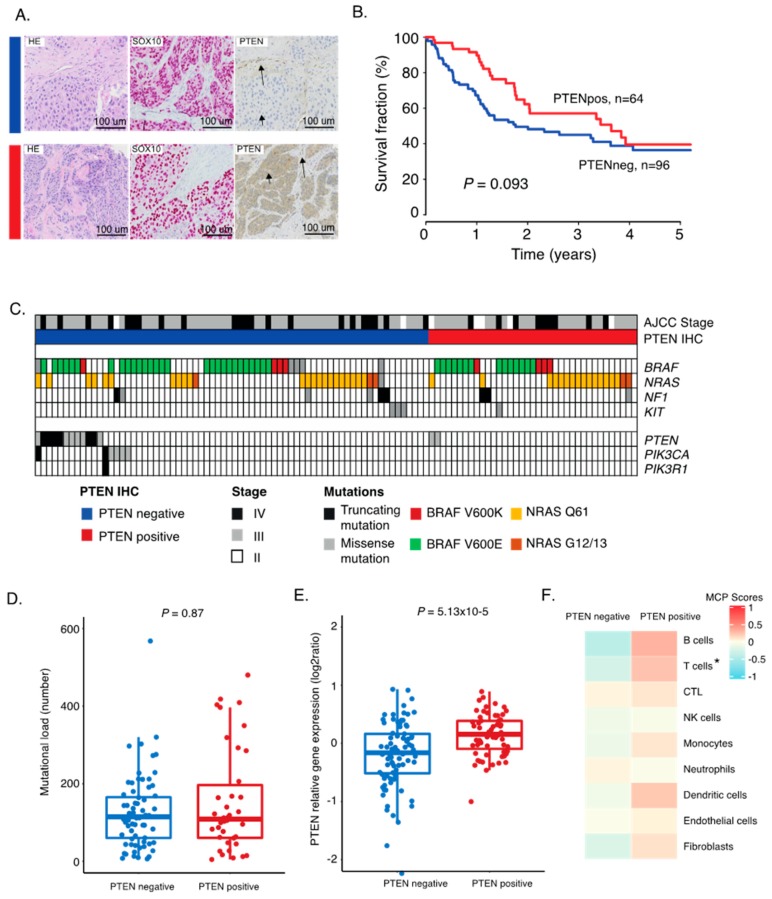
Characterization of PTEN expression groups in melanoma tumors. (**A**) Immunostaining of HE, SOX10, and PTEN on tissue microarray representative cores. Sections were taken consecutively. A PTEN-negative case and a PTEN-positive case are shown. Arrowheads indicate tumor cells, and arrows indicate non-tumor cells. (**B**) Kaplan–Meier survival analysis using log-rank tests of PTEN. (**C**) Mutational pattern of representative genes of the MAPK and PI3K pathways in PTEN-positive and -negative tumors. Twelve tumors in the PTEN negative group had *PTEN* mutation; six cases had *PIK3CA* mutation; and one harbored *PIK3R1* mutation. Among the PTEN-positive tumors, only two *PTEN* mutated tumors were found. (**D**) Mutational load across PTEN grouping. (**E**) Boxplot of gene expression of the *PTEN* gene between PTEN-positive and -negative tumors. *p*-values in boxplots were calculated using Wilcoxon analysis. (**F**) Average microenvironment cell populations (MCP) in PTEN-positive and PTEN-negative groups displayed in a heatmap. * FDR < 0.05. All others were non-significant.

Melanoma tumors from 169 patients were organized in tissue microarrays (TMA). The majority of the patients were diagnosed with a regional metastatic disease (55%), while 30% had a distant metastatic disease (Table 1). We used immunostainings to determine the PTEN protein status and utilized SOX10 as a melanoma cell marker. Thus, only SOX10-positive tumor cells were scored for PTEN expression status (Figure 1A). We found 59% lacking PTEN expression and 41% that had retained PTEN expression. No difference in gender or age at diagnosis based on PTEN status was observed. However, more advanced stage melanomas were PTEN-negative, and primary tumors were enriched in PTEN-positive cases (Table 1). Survival analysis showed that PTEN-positive tumors were linked to a better patient outcome (Figure 1B). This difference is likely not related to differences in treatment between groups, as we found no difference in treatment modalities between the PTEN groups (*p* = 0.8, Fisher’s exact test) However, when adjusting for stage, PTEN status was not an independent variable (*p* = 0.53, Cox regression). Furthermore, more mutations in PTEN were found in PTEN-negative cases than in positive cases (*p* = 0.13, Fisher’s exact test). Overall, somatic genetic alterations in any of the PI3K pathway genes were enriched in the PTEN positive cases (*p* = 0.016; Figure 1C). Interestingly, we found no difference in mutations in the MAPK pathway (*p* = 0.9, Fisher’s exact test). There was no difference in mutational load between PTEN-positive and -negative cases, suggesting that these tumors evolve independent of tumor genetic mechanisms (Figure 1D). When checking the level of expression of the PTEN gene in both PTEN immunohistochemistry (IHC) groups, not surprisingly, we confirmed a higher gene expression level in the PTEN-positive group of tumors (*p* = 5.13 × 10^−5^; Figure 1E). Overall, these results suggested that a significant fraction of metastatic melanoma tumors have lost their PTEN protein and that such cases are enriched in somatic mutations in the PI3K pathway.

### 2.2. Correlation between PTEN Alteration and Tumor Immune Microenvironment

Several molecular mechanisms explaining how tumor cells escape the immune system have been proposed [10,14,15,16]. To investigate the relationship between retained PTEN expression and immune cell presence, we used an antibody against CD8 to detect T-cells on the TMA. These cells were found to be either highly infiltrative, located in clusters, or completely absent in the melanoma tumor. We found that the presence of T-cells was associated with retained PTEN expression, supporting that PTEN may act as a regulator of immune escape (*p* = 0.003, Fisher’s exact test). We then investigated transcriptional patterns representing different immune cell subsets using the microenvironment cell populations-counter (MCP counter). This method allows the robust quantification of the absolute abundance of eight immune and two stromal cell populations in heterogeneous tissues from transcriptomic data [17]. Herein, both the T-cell and the cytotoxic T-cell signatures were downregulated in tumors lacking PTEN protein; however, all immune related signatures were generally downregulated in PTEN-negative cases (Figure 1F and Figure S1). Overall, this shows that PTEN alterations play a role in the tumor immune microenvironment by promoting immune evasion.

### 2.3. Inactivation of PTEN and Melanoma Cell Differentiation State Predicts Melanoma Survival

**Figure 2:**
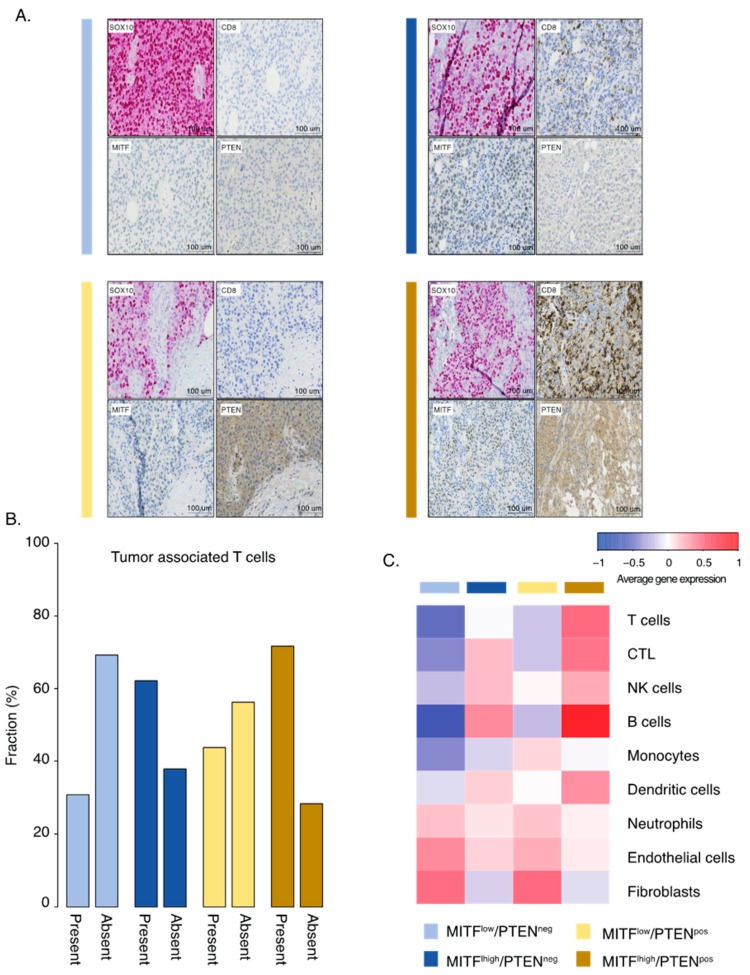
Immunological characterization of PTEN/MITF groups. (**A**) Representative immunostaining of SOX10, CD8, MITF, and PTEN. Sections were taken consecutively. (**B**) Bar plots of the fraction of present or absent tumor-associated T-cells using immunostaining in the MITF/PTEN groups. (**C**) Gene expression heatmap of immune cell populations using the MCP algorithm across the MITF/PTEN groups. Each column is the average score across samples belonging to the respective MITF/PTEN group.

**Figure 3:**
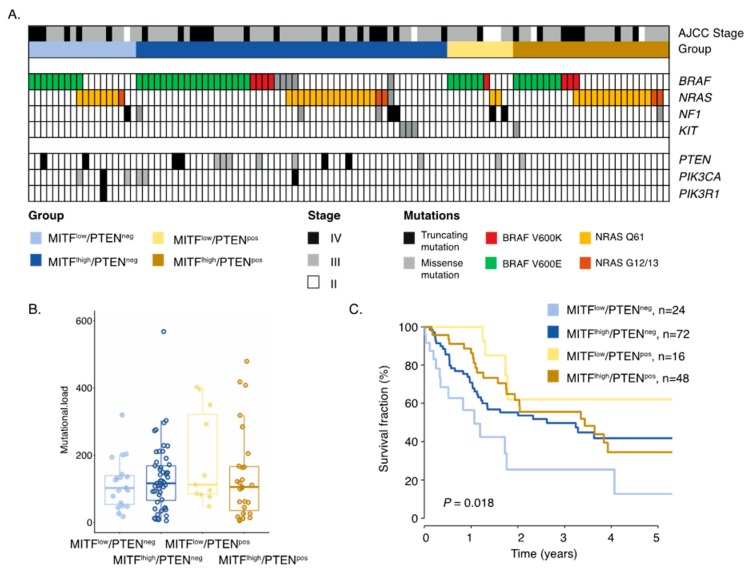
Molecular characterization of MITF/PTEN groups. (**A**) Mutational pattern of representative genes of the MAPK and PI3K pathways in MITF/PTEN groups. In MITF^low^/PTEN^neg^, two harbored *PTEN* mutation, three cases had *PIK3CA* mutation, and one had *PIK3R1* mutation. In the MITF^high^/PTEN^neg^ group, ten had *PTEN* mutation and one had *PIK3CA* mutation. The two other group had one *PTEN* mutated case each. (**B**) Mutational load across MITF/PTEN grouping using the number of mutations across 1500 cancer genes. *p* > 0.5, Kruskal–Wallis analysis. (**C**) Kaplan–Meier survival analysis using log-rank tests of MITF and PTEN markers combined. Survival data were missing for two cases in both MITF^low^/PTEN^neg^ and MITF^high^/PTEN^neg^ groups and five cases in the MITF^high^/PTEN^pos^ group.

It is well established that melanoma cells can exist in either MITF^high^ or MITF^low^ states. Such melanoma cells have different molecular properties and respond differently to therapy [9,18]. Thus, we analyzed the role of PTEN in MITF^low^ and MITF^high^ melanomas, respectively. In total, 42 cases were MITF^low^ and 127 were MITF^high^ by immunostaining; however, MITF status was independent of PTEN protein expression (*p* = 0.72, Fishers exact test). Nevertheless, we combined MITF and PTEN status, resulting in four different biological subgroups (Figure 2A). Indeed, only eight out of 26 (31%) MITF^low^/PTEN^negative^ (MITF^low^/PTEN^neg^) melanomas had tumor-associated CD8+ T-cells, while the corresponding results for MITF^high^/PTEN^positive^ (MITF^high^/PTEN^pos^) melanomas were 38 out of 53 (72%) (Figure 2B). We then went on to characterize these subgroups molecularly. Using the MCP immune gene signature algorithm, we found that MITF^high^/PTEN^pos^ melanoma tumors had the highest scores for T-cells, CTLs, NK cells, and B-cells, suggesting that such melanomas are more immunologically inflamed than others (Figure 2C). Next, we investigated mutations in the MAPK and PI3K pathways. No significant differences in *BRAF*, *NRAS*, or *NF1* mutations were identified between the groups, suggesting that these groups were independent of MAPK pathway mutations. Mutations in the PI3K signaling pathway were enriched in the two PTEN deficient groups, as expected (Figure 3A). Moreover, there was no difference in tumor mutational burden between the four groups, suggesting that these groups also evolved independent of tumor genetic mechanisms (Figure 3B). Intriguingly, we found that MITF^low^/PTEN^neg^ melanoma tumors were associated with a worse survival, and this was independent of stage (*p* = 0.1, multivariate Cox regression; Table S1; Figure 3C). Overall, we defined a group of melanomas with activated PI3K signaling and the loss of the melanocyte differentiation marker as having poor prognosis.

**Figure 4:**
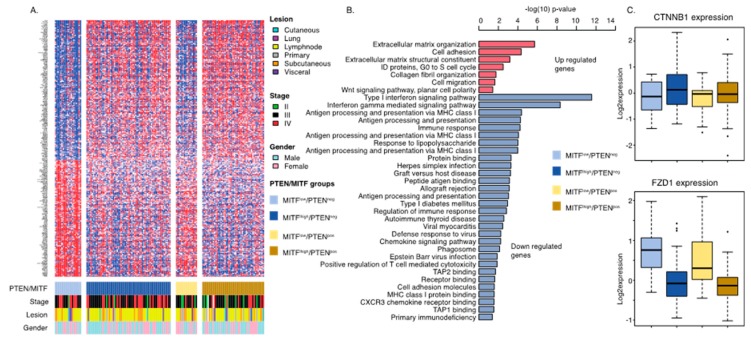
Transcriptional analysis of MITF^low^/PTEN^neg^ melanoma tumors. (**A**) SAM analysis identified genes (n = 276) characteristic of MITF^low^/PTEN^neg^ melanoma tumors. (**B**) Bar plot showing results from the gene ontology analysis using the 276 genes differentiating MITF^low^/PTEN^neg^ melanoma tumors from other melanomas. Notably, there are two cell adhesion molecule gene ontology terms. Genes downregulated (blue) include immune adhesion molecules, while upregulated genes include classical adhesion molecules such as N-cadherin. (**C**) Boxplots of the *CTNNB1* (beta-catenin) and *FZD1* gene expression value across the MITF/PTEN groups. CTNNB1 shows no significant difference (*p* = 0.24), while *FZD1* expression shows a significant difference (*p* < 0.0001).

As the MITF/PTEN groups were independent of tumor genetic mechanisms, we wanted to elucidate whether any underlying transcriptional differences could be discerned. In the survival analysis, we found that MITF^low^/PTEN^neg^ tumors were associated with poor survival (Figure 3C); hence, we were interested in determining a gene signature characterizing such melanomas. We found 279 significantly expressed genes (FDR = 0, fold change > 1.5; Table S2; Figure 4A) of which 152 were downregulated and 127 were upregulated in MITF^low^/PTEN^neg^ tumors as compared to other tumors. Using gene ontology tools, we found that immune related pathways, in particular pathways involved in antigen presentation, were enriched among downregulated genes (Figure 4B). In contrast, upregulated genes were enriched in pathways involved in cell adhesion and migration. Interestingly, we found that upregulated genes were also enriched in the Wnt signaling pathway, supporting previous reports demonstrating the importance of Wnt signaling in immune evasion [19]. Specifically, *FZD1* displayed overexpression in the MITF^low^ groups, while *CTNNB1* expression was equal across the groups (Figure 4C). Collectively, we identified that MITF^low^/PTEN^neg^ melanomas were characterized by a low frequency of lymphocytic infiltration, poor patient survival, and Wnt pathway activation.

### 2.4. Gene Signature of Dedifferentiated PTEN Inactivated Melanoma Predicts Response to MAPK Inhibition and Immune Checkpoint Therapy

**Figure 5:**
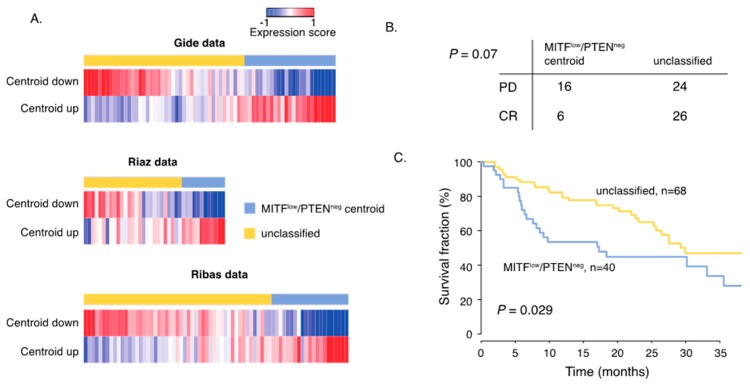
MITFlow/PTENneg gene signature predicts response to MAPK inhibitors and immune checkpoint blockade (ICB). (**A**) Heatmaps display the expression score of the MITF^low^/PTEN^neg^ centroid (up- and down-regulated genes in the centroid separately) in the ICB and BRAF/MEK inhibitor datasets. (**B**) Response data show the number of patients that developed PD (partial disease) or CR (complete response) to BRAFi/MEKi treatment in the two transcriptional groups. *p*-values were calculated using Fishers exact test. (**C**) Kaplan–Meier survival analysis using log rank tests of MITF^low^/PTEN^neg^ tumors and the unclassified group in ICB treated patients.

Although several treatment predictive biomarkers have been suggested, there is a medical need to further understand why some melanomas do not respond to therapy. It is also conceivable that there may be overlapping molecular features determining response to ICB and targeted therapy. Thus, we used the gene signature (n = 276 genes) characterizing the MITF^low^/PTEN^neg^ melanomas and created a centroid that was applied on gene expression data derived from patients receiving ICB or BRAF/MEK inhibitors [20,21]. The first cohort consisted of 32 patients exhibiting complete response (CR) from either Vemurafenib or Vemurafenib/Cobimetinib and 40 patients that displayed progressive disease (PD) during treatment [21] (Figure 5A). In total, tumors from 21 patients were classified (correlation (cor.) > 0.2) as MITF^low^/PTEN^neg^ of which 16 patients displayed PD; thus, there was an enrichment of patients with PD in the MITF^low^/PTEN^neg^ group (Figure 5B, *p* = 0.07, Fishers exact test). Next, we used two melanoma patient cohorts treated with ICB [20,21]. The first cohort included RNA sequencing data from pre-treatment tissue samples of 69 patients receiving either anti-PD1 monotherapy or anti-PD1/CTLA4 combination therapy. We found 25 cases having a centroid correlation > 0.2, suggesting high transcriptional similarity with MITF^low^/PTEN^neg^ tumors (Figure 5B). The second cohort consisted of 42 patients receiving anti-PD1 monotherapy [20]. Herein, 14 cases were classified as MITF^low^/PTEN^neg^. We then combined both cohorts and found that patients with MITF^low^/PTEN^neg^ classified melanomas were associated with inferior patient survival independent of study population (HR = 1.9; 95%CI 1.1-3.2, *p* = 0.03, multivariate Cox analysis, Figure 5C). Thus, we identified molecular features that corresponded to melanoma tumors resistant to currently available therapeutic regimens.

## 3. Discussion

During the last decade, the detailed understanding of the molecular mechanisms behind immune activation in cancer has changed the field of immunotherapy. On the other hand, it has been demonstrated that the dysfunction of the host’s immune system is one of the major ways by which tumors evade immune surveillance. This dysfunction can be caused by different host or tumor related factors [14]. The activation of the Wnt/B-catenin pathway is one of these factors, which is closely connected to the MITF “rheostat” model. According to this model, depending on the levels of its expression and activity, MITF signaling can result in both inhibition and promotion of melanoma growth. Very low levels of MITF or absence of MITF result in a population of completely arrested, potentially senescent cells. It is known that MITF expression can be regulated by canonical and non-canonical Wnt signaling, and thus, Wnt signaling can differentially affect each phase of the “rheostat”. Non-canonical Wnt signaling is more often found to be prevalent in MITF low cells [22].

The activation of the PI3K pathway through loss of PTEN is the second most frequent alteration associated with the non-T-cell inflamed phenotype in metastatic melanoma [4]. Several studies show that the loss of PTEN may mediate immune evasion by mitigating tumor-antigen cross-presentation, resulting in T-cell exclusion [16]. In this study, we aimed to explore PTEN alterations in our melanoma cohort and the correlation with the immune microenvironment and patient outcome. When comparing PTEN status with clinical features, such as age or gender, we concluded that PTEN loss was not dictated by either of these factors. However, we found that stage was a determinant factor when it came to PTEN status, as more advanced stage melanomas were more frequent among PTEN-negative and primary tumors were more frequent among PTEN-positive cases. Knowing that PTEN loss accumulates over the course of the disease, we hypothesized that the protein expression status of this protein could be linked to patient outcome. Not surprisingly, survival analysis showed that PTEN-positive tumors were linked to a better patient outcome, proving the crucial role of this tumor suppressor gene. This is in line with recent studies demonstrating that PTEN loss is associated with poor survival in stage III melanoma [4,23]. As expected, when analyzing further the specific PTEN alterations present in our cohort, more mutations were consistently found in PTEN-negative cases than in positive cases, supporting that genomic alterations led to loss of protein. Previously, genomic alterations in the *PTEN* and *BRAF* genes have been shown to co-occur, suggesting the loss of PTEN as a possible predicting factor of intrinsic resistance to BRAF inhibitor therapy [24]. As mentioned above, PTEN loss is described as being a driver factor of an immune evasion mechanism that results in a lack of T-cell tumor infiltration. This evidence led us to look at the correlation between PTEN status and T-cell infiltration in our cohort. We found that the presence of T-cells was associated with retained PTEN expression, supporting that PTEN may act as a regulator of immune escape [4,10]. To further characterize the influence that PTEN status has on the immune microenvironment, we found that transcriptional signatures representing T-cells and the cytotoxic T-cells were downregulated in tumors with PTEN protein loss. More importantly, the B-cell signature was also clearly downregulated in these tumors, which showed that PTEN loss might indirectly influence the function of other types of immune cells. This is of particular interest due to the presence of tertiary lymphoid structures that previously have been reported [25,26]. Tumors with tertiary lymphoid structures were highly inflamed and had improved prognosis and response to ICB. This opens the hypothesis that the combination of PI3K inhibitors and ICB may facilitate tertiary lymphoid structure formation. Dedifferentiation of melanoma tumor cells through the loss of MITF has been indicated as an immune evasion mechanism [9]. Thus, we hypothesized that tumors showing loss of both MITF and PTEN proteins simultaneously could correspond to an extreme immune-poor subtype. When combining tumors in four different groups according to the expression of both proteins, we corroborated our hypothesis, after observing that MITF^low^/PTEN^neg^ melanomas had lower counting scores for immune cell subsets than the MITF^high^/PTEN^pos^ group. As expected, this translated into a worse survival for the MITF^low^/PTEN^neg^ tumors. Interestingly, when searching for transcriptional signatures characterizing such tumors, we found that upregulated genes were enriched in the Wnt signaling pathway. This is concordant with the fact that Wnt signaling is well documented in immune evasion, and tumors with high Wnt signaling display lower immune cell infiltration [19]. Indeed, MITF^low^/PTEN^neg^ tumors were further characterized by downregulation of immune related genes. In particular, genes involved in antigen presentation were enriched among downregulated genes. Loss of MHC molecules has previously been described as an immune evasion mechanism and resistance to ICB [27], supporting our findings that MITF^low^/PTEN^neg^ tumors downregulated MHC related genes. Using the MITF^low^/PTEN^neg^ transcriptional signature on tumors from patients treated with BRAF/MEK inhibitors, we found that tumors resembling such tumors were more likely to have a progressive disease under treatment. This was concordant with previous studies describing that MITF^low^ melanoma cells are intrinsically resistant to BRAF inhibitors [5]. Furthermore, genomic alterations in the PI3K pathway such as *PTEN* mutations have been linked to acquired or intrinsic resistance to MAPK inhibitors [24]. Moreover, melanoma tumors with a transcriptional resemblance to MITF^low^/PTEN^neg^ tumors further displayed inferior survival following ICB. This supports that inactivation of PTEN may lead to immune evasion [4]. These results suggest that distinct molecular and transcriptional properties may predict the poor clinical benefit of both targeted therapy and ICB. Hence, understanding the underlying biology of melanoma states with distinct molecular properties and that display intrinsic resistance to different therapeutic regimens is crucial to identify novel ways of treating such melanomas.

## 4. Materials and Methods

### 4.1. Patient Material

This study was approved by the Regional Ethics Committee at Lund University (Dnr. 191/2007 and 101/2013). The sample cohort, representing a population-based retrospective collection (n = 169), was obtained at the Department of Surgery at Skåne University Hospital. Overall, the tumor cohort consisted of 108 regional lymph node metastases, 45 distant metastases, and 14 primary tumors or local recurrences. Five tumors were of unknown origin. This is a historic cohort collected between 2000 and 2012. Treatment information was available for 154 patients; 73 patients (47%) were untreated; and 81 patients (53%) were treated. Of the 81 cases, nine cases received targeted molecular therapy (mainly Vemurafenib), 35 were treated with immunotherapy (mainly interferon treatment), and 37 cases received chemotherapy. Treatment was initiated when patients had developed distant metastatic disease. As such, the cohort was suitable for prognostic studies. A summary of the patient characteristics is provided in Table 1. All subjects gave their informed consent for inclusion before they participated in the study. Data from different molecular are available on these tumors and shown in Figure S2.

### 4.2. Immunohistochemistry

Tissue microarrays (TMA) were constructed using on average of three 1 mm cores per tumor in an attempt to obtain a representative picture of the tumor. The tissue block was cut in four micrometer sections and then dried at 60 °C for one hour. The paraffin embedded sections were deparaffinized and pretreated in PT-Link (DAKO) with TRS (Target Retrieval Solution) buffer, pH 9. The following steps (except for the primary antibody stainings) were performed in the DAKO staining equipment (Autostainer plus) with Dako kit K8010 solutions: peroxidase block (5 min), EnVisionHRP-conjugated polymers (30 min), DAB substrate-chromogen solution (2 × 5 min), and counterstaining with hematoxylin (4 min). Between each step, the sections were rinsed with washing buffer. Finally, the sections were dehydrated and mounted with PERTEX mounting media (Ref. 00811) (Histolab). The primary antibodies used were all from Agilent/Dako: CD8 (M7103) in 1:100 dilution and MITF (Clone C5) in 1:400 dilution. PTEN was monoclonal (138G6, Cell Signaling Technology; dilution, 1:200; incubation, 2 h). SOX10 was performed in the clinical routine laboratory of Clinical Pathology at Skåne University Hospital by using the mouse monoclonal IgG1 (clone BC34, Biocare Medical) antibody. PTEN-positive and -negative cases were scored as described [28], by using a staining intensity score for tumor and non-tumor cells. PTEN status was recorded as a binary value, with PTEN-negative tumors having reduced to no immunostaining and PTEN-positive tumors having SOX10^+^ tumor cells stained. Overall, PTEN staining on the five TMA blocks is shown in Figure S3.

### 4.3. Bioinformatic and Statistical Analyses

Microarray expression data were generated using the Illumina HT12 array, previously used by Cirenajwis et al. [29], and have been deposited in Gene Expression Omnibus GSE65904.

The MCP signatures were used to investigate transcriptional patterns representing different immune cell populations. The MCP-counter is available as an R package. From a gene expression matrix, it produces for each sample an abundance score for CD3^+^ T-cells, CD8^+^ T-cells, cytotoxic lymphocytes, NK cells, B lymphocytes, cells originating from monocytes (monocytic lineage), myeloid dendritic cells, neutrophils, as well as endothelial cells and fibroblasts [17]. The genes included in each group were the following:

- T-cells: *CD28*, *CD3D*, *CD5*, *TRAT1*
- CD8 T-cells: *CD8B*, *CD8A*
- Cytotoxic lymphocytes: *EOMES*, *GNLY*, *KLRC4-KLRK1*
- NK cells: *KIR2DL3*, *KIR2DL4*, *KIR3DS1*, *NCR1*
- B lineage: *CD19*, *CD79A*, *CD79B*, *MS4A1*
- Monocytic lineage: *ADAP2*, *CSF1R*, *RASSF4*, *TFEC*
- Myeloid dendritic cells: *CD1A*, *CD1B*, *CD1E*, *CLEC10A*
- Neutrophils: *CEACAM3*, *CXCR1*, *CXCR2*, *FCGR3B*
- Endothelial cells: *CDH5*, *MMRN1*, *MMRN2*, *VWF*
- Fibroblasts: *COL1A1*, *COL6A2*

Mutation data were generated using a sequencing panel targeting 1550 cancer genes as previously described [10]. The ICB treated RNA-seq datasets [18,20] were downloaded from PRJEB23709 and https://github.com/riazn/bms038_analysis/tree/master/data, respectively, and datasets were processed as described previously [25]. The BRAF/MEK inhibitor treatment RNA-seq dataset [21] was downloaded from the respective Supplementary Table S2. EntrezGene IDs were converted to Gene Symbols; protein-coding genes were kept; samples were quantile-normalized; and the data were log-transformed using log2(data + 1).

To determine genes with significantly different expression between MITF^low^/PTEN^neg^ tumors and other melanomas, we used significant analysis for microarray (SAM). Genes with false discovery rate (FDR) = 0 and foldchange > 1.5 were included in the signature. Gene Ontology analysis was performed using the online DAVID software [30]. To create the centroid, the average expression value across MITF^low^/PTEN^neg^ tumors was obtained for each of the 276 genes. The centroid was subsequently applied to external datasets by matching on gene symbol and calculating the Pearson correlation (cor.) for each sample against the centroid. All samples with a cor. >0.2 were classified as transcriptionally similar to MITF^low^/PTEN^neg^ tumors.

Fisher’s exact test was used for comparison of categorical variables, and Pearson correlation was used for comparison of numerical variables. The Kruskal–Wallis test was used for the association between numerical and categorical variables. Cox regression from the survival package was used for survival analyses. All tests were two-sided. Bioinformatical analyses were done with R software. Boxplots are depicted with the center line representing the median, the box limits representing the lower and upper quartiles, and the whiskers extending to the most extreme values within 1.5 × IQR.

## 5. Conclusions

In conclusion, in this study, we analyzed the clinical significance of PTEN protein alterations in a metastatic melanoma cohort. Moreover, we described that inactivation of PTEN in conjunction with loss of melanocyte differentiation features led to a highly aggressive melanoma phenotype with molecular properties rendering it resistant to targeted- and immuno-therapy.

## Figures and Tables

**Table 1:** Clinical features of the 169 melanoma cases in correlation with PTEN status. *p*-values from Fisher’s exact test, not adjusted for multiple testing.

Feature	Entire Cohort (n = 169)	PTEN+ (n = 69)	PTEN− (n = 100)	*p*-Value
Gender n (%)				1
Male	98 (58)	39 (57)	59 (59)	
Female	69 (41)	28 (41)	41 (41)	
NA	2 (1)	2 (3)	-	
Age at diagnosis median (range)	65 (22–91)	65 (22–91)	65 (30–88)	0.54
Stage				0.003
II	18 (11)	13 (19)	5 (5)	
III	99 (59)	41 (59)	58 (58)	
IV	50 (29)	13 (19)	37 (37)	
NA	2 (1)	2 (3)	0	
Lesion type				0.004
Lymph node	108	36	72	
Subcutaneous	35	16	19	
Visceral	10	4	6	
Primary tumor	14	11	3	
NA	2	2	-	
Histological subtype				0.5
Unknown primary n (%)	26 (15)	9 (13)	17 (17)	
SSM	35 (21)	11 (16)	24 (24)	
NM	57 (34)	24 (35)	33 (33)	
Other	14 (8)	5 (6)	9 (9)	
NA	37 (22)	20 (29)	17 (17)	

SSM, superficial spreading melanoma; NM, nodular melanoma; NA, not available.

## Supplementary Materials

The following are available online at https://www.mdpi.com/2072-6694/12/3/742/s1: Figure S1: MCP immune scores in melanoma tumors, Figure S2: Flowchart of different data levels included in the study, Figure S3: PTEN staining in the five tissue microarray (TMA) blocks, Table S1: Multivariate cox regression model analysis of MITF/PTEN groupings adjusted for stage of disease, with hazards ratios and confidence intervals, Table S2: Centroid used in the classification.
