# Supplementary material for: The Role of PTEN Loss in Immune Escape, Melanoma Prognosis and Therapy Response
Source: Cancers (Basel). 2020 Mar 21;12(3):742. doi: 10.3390/cancers12030742 (PMC7140048; doi:10.3390/cancers12030742)
Supplement: Supplementary file 1 [file cancers-12-00742-s001.pdf]

Supplementary Materials: The Role of PTEN Loss in Immune Escape, Melanoma Prognosis and Therapy Response

Rita Cabrita, Shamik Mitra, Adriana Sanna, Henrik Ekedahl, Kristina Lövgren, Håkan Olsson, Christian Ingvar, Karolin Isaksson, Martin Lauss, Ana Carneiro and Göran Jönsson

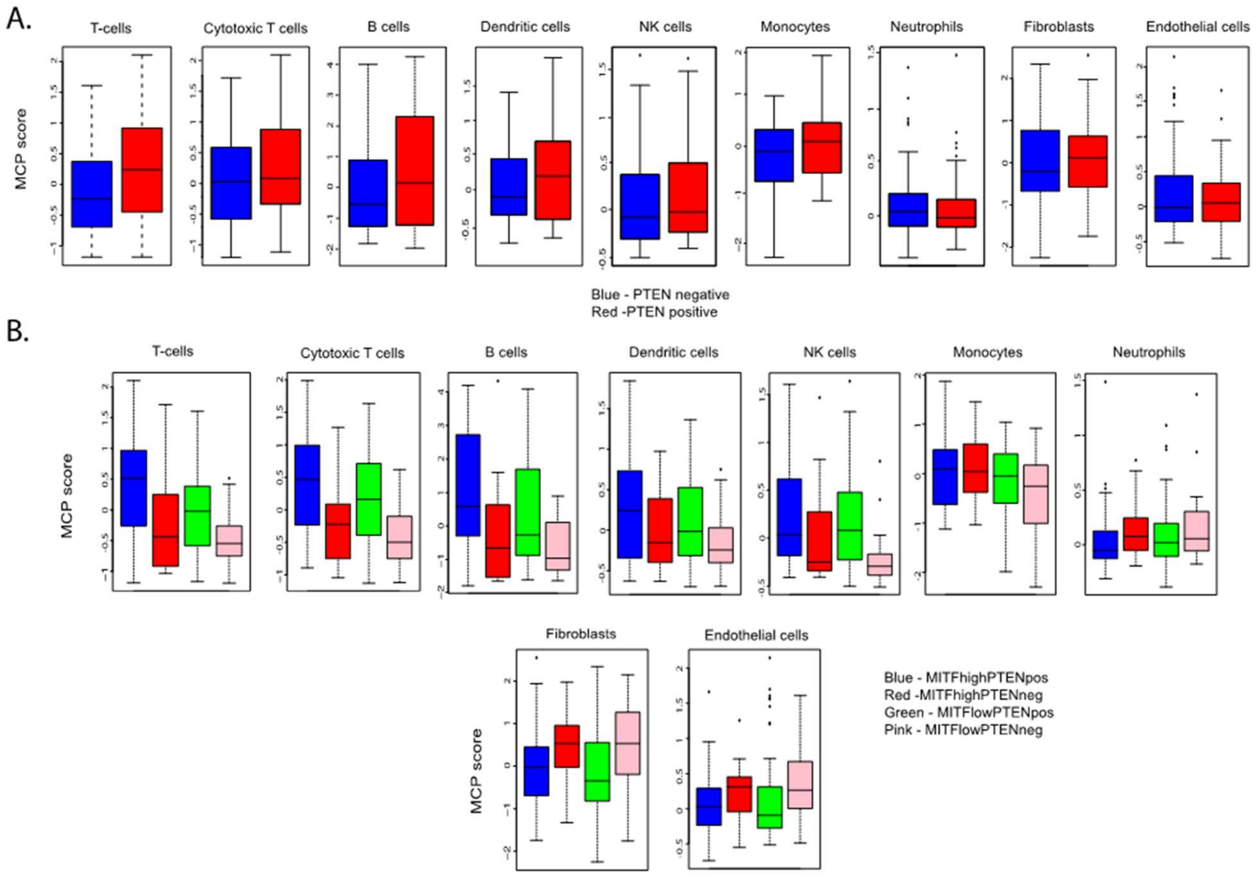

Figure S1. MCP immune scores in melanoma tumors. (**A**) MCP scores in PTEN negative (blue) and PTEN positive (red) tumors. (**B**) MCP scores in groups based on PTEN and MITF protein.

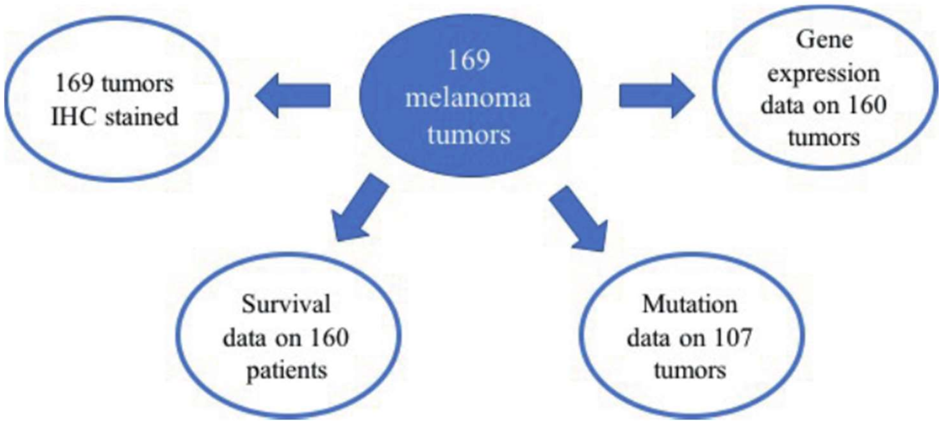

Figure S2. Flowchart of different data levels included in the study.

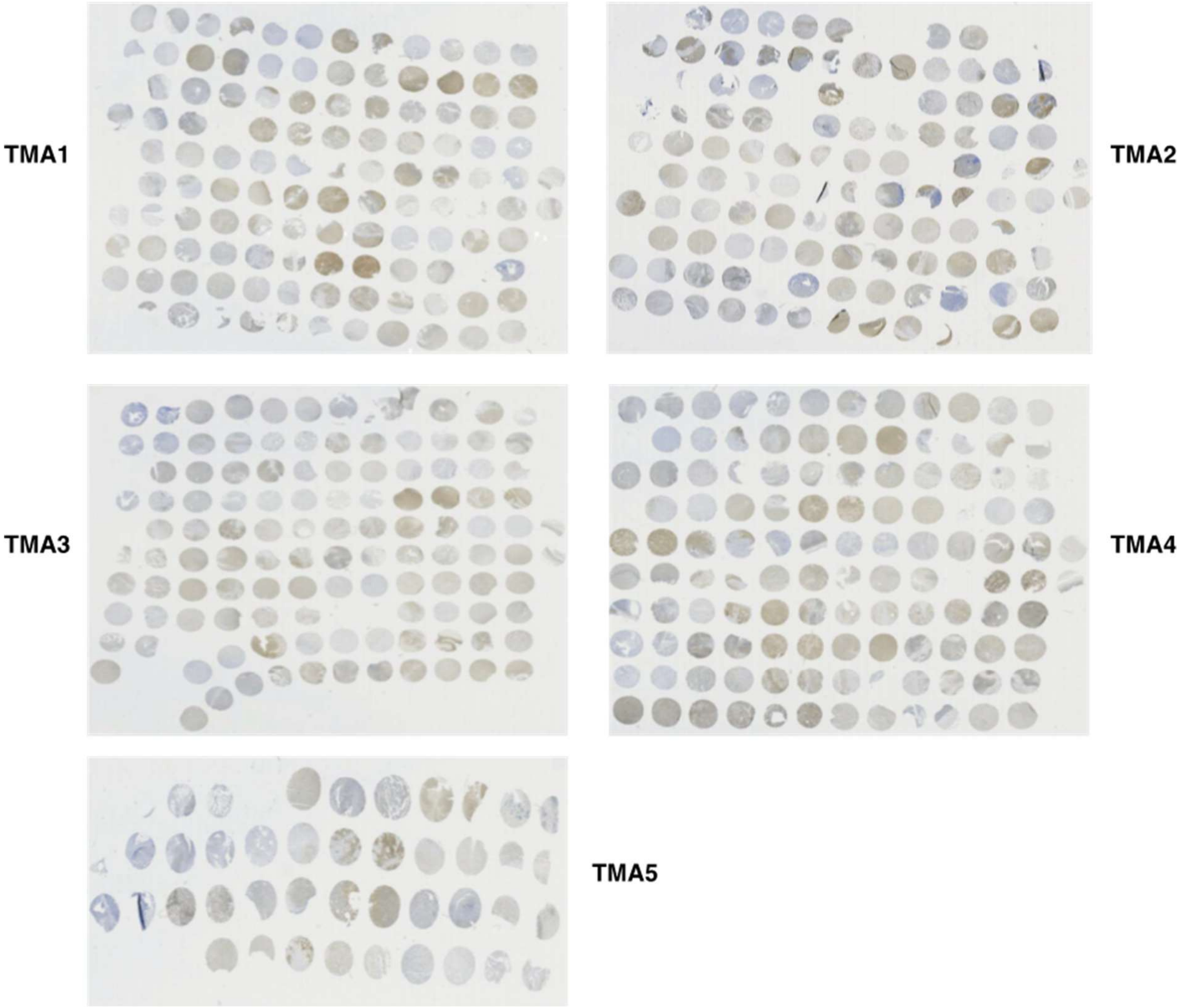

Figure S3. PTEN staining in the five tissue microarray (TMA) blocks.

Table S1. Multivariate cox regression model analysis of MITF/PTEN groupings adjusted for stage of disease, with hazard ratios and confidence intervals.

Group	HR (CI)	*p*-Value
MITF/PTEN		
$MITF^{high}PTEN^{pos}$	1	-
$MITF^{high}PTEN^{neg}$	0.96 (0.56, 1.66)	0.89
$MITF^{low}PTEN^{pos}$	0.90 (0.34, 2.41)	0.84
$MITF^{low}PTEN^{neg}$	1.76 (0.89, 3.51)	0.11
Stage		
II	1	-
III	1.89 (0.26, 13.95)	0.53
IV	7.29 (0.99, 53.73)	0.05

Table S2. Genes and log2 expression value included in the centroid.

Genes down in MITF^{low}/PTEN^{neg}	Value (log2 Expression)	Genes up in MITF^{low}/PTEN^{neg}	Value (log2 Expression)
ITGB7	−0.6637874	CADM1	1.646150435
CD79A	−1.1278003	DACT3	1.284409212
TRIB1	−0.627541	SLIT2	1.069307448
CXCL10	−0.9425556	PCDH18	1.272287668
HLA-C	−0.6630066	DIAPH3	1.049453117
PLEK	−0.8497991	C5orf13	1.463647947
SDCBP	−0.6168393	EFNB2	1.037998007
VNN2	−0.7245285	LOC730994	0.816222521
CSTB	−0.6004923	HOXB5	0.890360545
CD8A	−0.9936763	MOXD1	1.464179261
ANKRD22	−0.6451211	PCDH17	0.945373709
CD52	−1.1162775	PHLDB2	0.937604318
NCF1	−0.6778936	CDH2	1.690163862
BHLHB3	−0.748998	DDAH1	0.904631728
HSD17B14	−0.6270172	POSTN	1.497294478
PARP10	−0.590338	ID3	1.086589022
BACE2	−0.7382248	FAM46A	0.87567871
IRF8	−0.9998088	SBK1	1.009141014
GMPR	−0.7436598	UBE2E3	0.702786405
UPP1	−0.6648916	ERRFI1	1.163913307
BIRC3	−0.9908605	NTM	1.054703811
PTPLA	−0.7938529	PMEPA1	1.072653617
SOX13	−0.5899106	ANGPT1	1.237634849
ROPN1B	−0.8454191	PCOLCE	1.201493612
TBC1D7	−0.7636963	FZD1	0.747943092
CCL5	−0.9803421	PFTK1	0.727311372
SNCA	−0.8087163	SPRY2	0.991593274
BCL2A1	−0.6826054	CDK6	0.99478154
SLC11A2	−0.6300387	VCAN	1.210859619
NAPSB	−1.0304074	DPYSL3	0.992375697
MAZ	−0.5829541	H19	1.385217542
ISG20	−1.0010139	TUSC3	1.081069528
FBXW5	−0.6088084	EDNRA	0.748145714
TAPBP	−0.6346449	FZD2	0.640606599
RAB32	−0.6490322	LMNB2	0.699635474
INPP4B	−0.723364	FLJ21986	0.98835468
SWAP70	−0.6226537	ANTXR1	0.773896172
MICAL1	−0.6903372	LOC100132901	0.628405303
PHF15	−0.6475682	PXDN	1.09475001
BIRC7	−0.9480172	MLLT11	1.107994167
UBE2L6	−0.6231672	S1PR3	0.701131508
RARRES3	−0.9509742	ATP2B1	0.690079064
LYN	−0.7620077	CLIP3	0.842692443
HCG4	−0.7113144	RBBP4	0.641617936
GSDMD	−0.6139003	SPON1	1.228882684
RAC2	−0.9906747	CBX2	0.809775067
SLC9A3R1	−0.6598906	HOXA5	0.87906882
RUNX3	−0.9927214	LPHN2	0.959883606
FAM69A	−0.6461069	DKK3	0.96728898
FCRLA	−1.2602482	LOC100134134	1.132476535
ST3GAL6	−0.726269	IGFBP2	1.175978716
IRF1	−0.855961	LOX	0.884465254

ECHDC3	−0.6181085	FOXC1	0.816302065
CD38	−0.7704543	PAM	0.799031548
SIGIRR	−0.5547153	LIMCH1	1.012433761
ROPN1	−0.9194562	CPXM1	0.789543917
FYB	−0.9039163	ZNF286C	0.591466564
SNX10	−0.6588212	LOC730525	0.964789048
HCST	−0.9173861	LAMB1	0.836327667
HERC5	−0.8169465	KLF12	0.635473272
OAS2	−0.9269088	LOC730101	0.552153478
MCOLN2	−0.7311837	BCAT1	0.86543584
APOE	−0.8097618	UBA52	0.71648786
IFIH1	−0.6855511	ZNF84	0.57808192
SAT1	−0.5598643	LOC100131139	0.746655149
RAB38	−1.0610584	TSPAN5	0.759215173
CD79B	−1.0782803	TMTC4	0.660299277
SOD2	−0.7568718	TCF12	0.670699942
GNPTAB	−0.5581993	AUTS2	0.785922266
PSMB9	−0.7884907	ID1	0.782572387
TMEM140	−0.7171343	TRIL	1.002751536
GZMB	−1.1113204	KDM5B	0.592819139
HPS1	−0.5510096	OLFML2A	0.657865462
RPS6KA5	−0.5670544	LOC653344	0.557543286
MFSD6	−0.654256	LRP5	0.677173513
PRKCB1	−1.0735466	EFEMP2	0.795275881
QPCT	−1.3166014	ZNF512	0.539659589
IFI27	−1.2217255	EXTL2	0.626539861
RGS10	−0.7862767	KDELC1	0.58089505
GSTO1	−0.6408478	GPC6	0.539021279
CXCL9	−1.2573445	TRO	0.739533058
NKG7	−1.0087833	TMEM47	0.877205827
PLXNC1	−0.7586767	LOC100133760	0.610353211
RAB27A	−0.7035185	RBP1	0.904180363
KAT2B	−0.7054284	RCAN2	0.815825294
SLC45A2	−1.3923654	ZNF395	0.466799304
ADAMDEC1	−1.2114369	COL8A1	1.104412183
HIGD1A	−0.5175034	LOC645166	0.708179748
IL18BP	−0.9325305	TLE4	0.855509022
GPNMB	−0.8294207	RGS4	0.808223983
PHACTR1	−0.89301	ENC1	0.70166635
CD3D	−1.3203313	SOX4	0.746289566
FAM65B	−0.9658844	PEG10	0.912269898
GBP5	−0.977453	LAMC1	0.640956415
PARP9	−0.6920852	PKIA	0.800216308
HLA-B	−1.035889	CNPY4	0.526343004
LOC390557	−0.711938	CENPV	0.637370896
CD48	−1.3089793	LOC728732	0.588492945
HCP5	−1.1201781	SSFA2	0.591275715
HLA-F	−0.9897719	FSCN1	0.845168008
TRIM63	−1.0193057	MEIS2	0.698440229
PLA1A	−1.3654242	TCF3	0.511660304
TYSND1	−0.5696128	SOCS2	0.711013533
CXCL13	−1.3775115	ZNF532	0.661004424
MGC13057	−0.8628089	NR4A2	0.792124417
CABLES1	−0.9646213	SKP2	0.550286352
MCOLN3	−1.1803748	STK32B	0.771656854
HRK	−0.9979237	COL16A1	0.57506291

OSTM1	−0.7563323	TMSB15A	0.974167792
ASB9	−0.7821446	SALL2	0.523090784
FAIM3	−1.2771405	SYT11	0.685722901
VAT1	−0.8184453	SMA4	0.490970275
ARL6IP5	−0.5472999	COL5A1	0.915868645
S100A1	−1.2352086	ITGB5	0.759491207
GYG2	−1.1467153	ESM1	0.802307819
LY96	−0.8734827	ENAH	0.536317296
CD44	−0.8390348	COL12A1	0.736125394
HLA-E	−0.8544589	LOC645385	0.519091808
FBXO6	−0.7278542	PRDM8	0.58278025
HLA-G	−0.9261851	EMILIN1	0.681825454
MITF	−1.0316963	PRICKLE1	0.673123973
RTP4	−0.7450338	COL5A2	0.880003949
SERPINB1	−0.765538	CPE	0.766764884
CBR3	−0.7069554	SDC2	0.812521025
ST6GALNAC2	−1.0839479	HNRPA1L−2	0.564260655
MYO5A	−0.8894011	PRICKLE2	0.62877922
PSMB8	−0.8434825	SPRY1	0.757695396
PRKCD	−0.7213112		
CEACAM1	−1.3066298		
IRF4	−1.0175048		
IFI35	−0.8226885		
STX7	−0.7898291		
TRPV2	−0.9233402		
CHCHD6	−1.3239017		
CASP1	−1.1742113		
STAT1	−1.0606008		
P2RX7	−0.9106965		
LYST	−1.1111641		
CAPN3	−1.5022855		
MBP	−1.1579674		
PIR	−1.1636714		
TAP1	−1.0964007		
LGALS3	−1.1585214		
TNFRSF14	−1.0275618		
WARS	−1.182983		
GPR56	−1.3043394		
UBD	−1.6572527		
RENBP	−1.1237332		
D4S234E	−2.0144985		
TYR	−2.3769119		
SILV	−2.8824723		
MLANA	−2.3966163		

© 2020 by the authors. Licensee MDPI, Basel, Switzerland. This article is an open access article distributed under the terms and conditions of the Creative Commons Attribution (CC BY) license (http://creativecommons.org/licenses/by/4.0/).
